# Ingroup Empathy, Help, and Blame After Anti-LGBT+ Hate Crime

**DOI:** 10.1177/08862605231200212

**Published:** 2023-09-13

**Authors:** Jenny L. Paterson, Mark A. Walters, Lisa Hall

**Affiliations:** 1 Northumbria University, Newcastle upon Tyne, UK; 2 University of Sussex, Brighton, East Sussex, UK

**Keywords:** hate crime, LGBT+ victimization, ingroup empathy bias, victim blame, helping intentions

## Abstract

Crimes motivated by hatred toward a person’s sexual orientation or gender identity typically cause greater physical and emotional harm than comparative crimes not motivated by hate. Compounding these impacts, hate crime victims receive less empathy, less support, and are blamed more for their victimization both by society in general and by criminal justice agencies. However, as hate crimes are the epitome of intergroup hostility, the crimes are also likely to engender an ingroup empathy bias in which fellow LGBT+ people provide greater empathy to hate crime victims, potentially motivating greater support and reducing victim blaming for these particularly marginalized victims. Across three studies, we examined LGBT+ participants’ empathic reactions to hate crime victims, along with their willingness to help victims and blame victims. In the Pilot Study (*N* = 131) and Study 1 (*N* = 600), we cross-sectionally showed that indirect experiences of hate crimes predicted a stronger LGBT+ identity which, in turn, was associated with greater empathy that predicted greater willingness to help victims and blame the victim less. In Study 2 (*N* = 657), we experimentally manipulated the motivation of a crime (hate vs. non-hate) and the group membership of the victim (ingroup-LGBT+ vs. outgroup-heterosexual) and found that crimes that had one or more group elements (i.e., involved an ingroup member and/or was motivated by hate) elicited greater empathy that, in turn, increased the willingness to help the victim and reduced victim blaming. Together, the findings provide cogent evidence that LGBT+ communities respond to anti-LGBT+ hate crimes with overwhelming empathy, and this ingroup empathy bias motivates helping behaviors and reduces victim blame, thereby buffering the marginalizing consequences of hate crimes. Policy implications include acknowledging and harnessing the importance of shared identities when practitioners and criminal justice agencies respond to anti-LGBT+ hate crimes.

Crimes motivated by, or which demonstrate, hatred or prejudice toward a person’s sexual orientation or gender identity typically cause greater physical and psychological harm than comparable crimes not motivated by hate (Home Office, 2020; Lantz & Kim, 2019). Consequently, victims of anti-LGBT+ hate crimes are more likely to report higher levels of depression, anger, and anxiety, and require more than twice as long to overcome their victimization than victims of comparable crimes (Fetzer & Pezzella, 2019). Providing supportive environments for victims of hate crime, then, is clearly essential for their recovery.

As trust and confidence in formal support organizations (e.g., criminal justice agencies) remain relatively low due to historical and ongoing marginalization (Feddes & Jonas, 2020; Walters, Paterson, Brown, et al., 2020), many victims of hate crime look to their “ingroup” communities for help and support (Haynes et al., 2023; Lockwood et al., 2022). Within LGBT+ communities, emerging evidence suggests that hate crime victims are generally met with considerable empathy (Paterson et al., 2019b). Yet, relatively little is known about community members’ willingness to proactively support hate crime victims in the aftermath of targeted attacks.

Conversely, research also suggests that these same victims can sometimes be blamed for their victimization by other LGBT+ people (Bell & Perry, 2015; Paterson et al., 2019b). Victim blaming has potentially negative consequences for victims’ emotional recovery as it feeds into social processes that lead to LGBT+ people experiencing internalized shame (Herek, 2004). Understanding when and why victims are supported or blamed for their experiences of victimization is of central importance to establishing measures that can effectively address the harms caused by anti-LGBT+ hate crimes.

This article reports extensive findings from three independent quantitative studies using large cross-sectional and experimental data examining ingroup victim support and blame in relation to LGBT+ hate crime. Using complementary and interdisciplinary literature, our research is the first to quantify the *extent* to which LGBT+ communities support or blame victims in the aftermath of hate crimes, as well as examine *why* support is offered or blame is apportioned. In so doing, we offer new evidence on the community dynamics of hate crime that has important implications for policy and practice.

## Group-Based Reactions to Group-Based Hostility

Social categorization theory (SCT; Turner et al., 1987) and the related intergroup emotions theory (IET; Smith, 1993) provide a useful framework for understanding the widespread impacts of hate crime and the reactions toward its victims (Paterson et al., 2019a). According to this perspective, when individuals identify—or *categorize* themselves—as members of social groups (e.g., as part of the LGBT+ community), they begin to react to their social environment in ways that not only reflect their individual experiences of the world but their collective ones too. This cognitive and affective shift from “I” (the individual) to “us” (the group member) has significant implications for how people appraise and respond to intergroup events. Attacks against other ingroup members, for example, can be felt and responded to as an attack on all group-identified individuals, even when these individuals are not directly involved in the incident (Mackie & Smith, 2015).

Hate crimes are particularly likely to be perceived as an attack against the ingroup because they (typically) target two central facets of the ingroup: they attack people who make up the ingroup *and* they symbolically target the overarching group identity. First, by targeting LGBT+ *ingroup members*, hate crimes are unambiguous attacks on a specific individual with a specific identity. Second, the public expression of anti-LGBT+ hate sends a clear message of intolerance and hatred toward the entire group’s *identity*, thereby affecting all those who also identify as LGBT+ (Paterson et al., 2019a). Accordingly, by targeting both ingroup members (i.e., individuals) and the LGBT+ identity (i.e., group), hate crimes increase the saliency of the sexual and gender identity which, in line with SCT and IET, is likely to elicit a common group-based response from LGBT+ people (Paterson et al., 2019a).

Illustrating these group-based reactions in the aftermath of anti-LGBT+ hate crimes, research has previously found that the number of hate crime victims known to an individual (defined here as *indirect experiences* with hate crimes, and sometimes referred to as *vicarious victimization*) is positively associated with depressive symptoms (Wenger et al., 2022), feeling more vulnerable and threatened, but also more empathy toward victims (Paterson et al., 2019b). Crucially, showing that these responses are group based and not simply a consequence of feeling more empathic toward *all* victims, an experiment showed that LGBT+ people feel more threatened and angry when reading about a homophobic hate crime compared to a similar crime not motivated by hate (Paterson et al., 2019a, Study 1). Furthermore, highlighting the importance of the hate motivation toward identity (rather than a specific victim), respondents reported a stronger LGBT+ identity and felt significantly angrier and more anxious when reading about an ingroup member in a hate crime than when the ingroup member was attacked in a case of mistaken identity (Paterson et al., 2019a, Study 2).

Key to the present research, across the two experiments hate crime victims elicited significantly greater empathy than both an outgroup victim (Paterson et al., 2019a, Study 1) and an ingroup victim attacked in a case of mistaken identity (Paterson et al., 2019a, Study 2). Although the research did not examine other reactions toward the victim (e.g., blame) or the inclination to help the victim in the studies, these findings showed that having indirect experiences with anti-LGBT+ hate crimes clearly primed participants’ LGBT+ identities and provoked group-based responses, including greater ingroup empathy, which were likely to influence how LGBT+ people reacted to hate crime victims.

## Intergroup Empathy Bias and its Implications for Helping and Blaming Victims

The previous finding that LGBT+ people report greater empathy for victims of hate crimes fits well with a wealth of research into *intergroup empathy bias*, which shows that people feel greater empathy for ingroup members over outgroup members in numerous contexts and across many different groups (Cikara et al., 2011; Vanman, 2016). Drawing on IET (Mackie & Smith, 2015), this ingroup empathy bias likely occurs because sharing an identity with a victim promotes cognitive and emotional bonds with other ingroup members, thereby facilitating a greater understanding of the victim and, crucially, feeling *as* and *for* that person—both of which are central characteristics of empathy (Batson & Ahmad, 2009).

As the empathy-altruism hypothesis suggests that empathy is a strong and reliable predictor of helping behaviors (e.g., providing emotional and financial support, Batson, 1991), this intergroup empathy bias has significant implications for understanding people’s willingness to support ingroup (vs. outgroup) members. Stürmer et al. (2006), for example, provide experimental evidence showing that when participants read about a person in need, they report more empathy for the person if they believe they are a fellow ingroup (vs. outgroup) member. This empathy then motivates individuals to provide more help to the ingroup member than the outgroup member—even when the groups are based on trivial, randomly assigned, meaningless identities. Such *ingroup* empathy-motivated help, furthermore, is particularly important because individuals tend to be more receptive to help from ingroup (vs. outgroup) members, and the help is often more effective (Nadler, 2002; Selvanathan et al., 2020).

Extrapolating these findings to hate crime contexts implies that LGBT+ individuals will feel more empathy for victims targeted for their (shared) LGBT+ identity and this greater empathy will motivate them to proactively support the recovery of these ingroup victims. Nevertheless, intergroup empathy bias also has potential effects on victim-blaming tendencies. There is substantial evidence suggesting that victims of all crimes are sometimes held responsible for their victimization (Bulman & Wortman, 1977), and hate crime is no exception (Erentzen et al., 2021). However, the extant literature on victim blaming in the aftermath of hate crimes is somewhat mixed in its conclusions. Some have found that victims of hate crimes are blamed *less* than victims of non-hate crimes (Rayburn et al., 2003; Saucier et al., 2010), while others have found that hate crime minority group victims elicit *less* empathy (Godzisz & Mazurczak, 2022) and are blamed *more* (Study 1, Plumm et al., 2014). Others suggest that blame is further contingent on other factors such as victim passivity (Erentzen et al., 2021), type of activity the victim is engaged in (Godzisz & Mazurczak, 2022), consumption of alcohol (Godzisz & Mazurczak, 2022), and location of crime (Plumm et al., 2010).

Complicating the picture further is that the research above predominantly focuses on outgroup members’ reactions to hate crimes. While these views are important in so much as they may be indicative of broader societal beliefs and even jurors’ attitudes (Plumm et al., 2014), blame attributed by *ingroup* members is likely to be different and have distinct consequences for the victim and community. Notably, because hate crimes are an attack on a shared identity, they are likely to be particularly threatening to other group members who may feel that they could be attacked next (Bell & Perry, 2015). Contrary to the research on ingroup empathy and its links to greater levels of support, there is still reason to believe that some group members will seek to alleviate this feeling of threat and increase their own sense of safety by engaging in ingroup victim blaming. For example, LGB participants interviewed in Bell and Perry’s (2015) qualitative study discussed how they sometimes assume victims have somehow provoked an attack, or that the victim could have avoided an assault by curbing their (effeminate) behaviors. Although others in the focus group were quick to challenge these insinuations of blame, it is clear that ingroup victim blame is evident among some individuals post-anti-LGBT+ hate crime incidents (Paterson et al., 2019b). Furthermore, because perceptions and reactions from ingroup members are particularly valued by ingroup members (e.g., Tajfel, 1978), this ingroup blame is likely to be especially harmful to victims who may internalize the blame. This can lead to characterological self-blame where the victim feels that faced with the situation again they could, or importantly should, have behaved differently (Noelle, 2009). This is illustrated by an interviewee in one anti-LGBT hate crime study who reflected “I am the one who feels ashamed because the inference is that they abused me in this way because of my body language or the way in which I looked at them . . .” (Dick, 2008, p. 31).

A potential mitigating factor of this ingroup victim blaming is the intergroup empathy bias found in reactions to anti-LGBT+ hate crimes. On the one hand, LGBT+ people are likely to feel their identity and physical safety are threatened when hearing of an anti-LGBT+ hate crime (vs. non-hate crime: Paterson et al., 2019a), and this is likely to elicit insinuations of blame in a possible effort to reduce these feelings of threat (Bell & Perry, 2015). On the other hand, LGBT+ people are likely to be more empathic toward hate crime victims and ingroup members in general because of their shared identities (Paterson et al., 2019a; Walters, Paterson, McDonnell, et al., 2020). This empathy, in turn, is likely to reduce ingroup victim blaming. In fact, this has been observed in sexual harassment contexts, where empathy for victims has been shown to strongly predict less victim blame (Bongiorno et al., 2020). Furthermore, because ingroup empathy is extremely pronounced in anti-LGBT+ hate crimes (Paterson et al., 2019a), it is likely to be a potent factor in reducing victim blaming for LGBT+ (vs. non-LGBT+) victims and those targeted in anti-LGBT+ hate (vs. non-hate) crimes.

## The Impact of Worldviews

Although group identities are central to understanding reactions toward group-based attacks (Paterson et al., 2019a), there is considerable evidence to suggest that individuals’ worldviews also dictate how people react to crime victims. One of the most widely studied factors is just world beliefs (JWB; Lerner, 1980). This worldview asserts that the world is fair and predictable and that people generally get what they deserve. It has many psychological benefits including providing a sense of stability and control over one’s life which enables individuals to plan and achieve long-term goals. However, as this worldview assumes that only bad things happen to bad people, it is incompatible with acknowledging that victims can be completely innocent, and so promotes victim blaming in a wide range of contexts including rape and domestic abuse (see Hafer & Sutton, 2016 for a review). Considering its well-established influence on reactions to victims, especially in terms of victim blaming, we include it here as a covariate to provide a stringent examination of whether group-based considerations (hate motivation and identity) explain reactions to hate crime victims above and beyond this well-known predictor of reactions toward victims in general.^1^

## Present Research

The program of research comprised three interrelated studies examining LGBT+ participants’ empathic reactions to hate crime victims, along with their willingness to help victims and blame victims. In the first two studies (Pilot and Study 1), we cross-sectionally tested whether indirect experiences of hate crimes predicted a stronger LGBT+ identity which, in turn, was hypothesized to be associated with greater empathy that predicted greater willingness to help victims and less victim blame. Building upon this, in Study 2, we experimentally manipulated the motivation (hate vs. non-hate) and victim (ingroup vs. outgroup) of a crime to assess how these group-based characteristics influenced reactions toward the victim. We expected that crimes that had one or more group element (i.e., involved an ingroup member and/or were motivated by hate) would elicit greater empathy which would, in turn, increase the willingness to help the victim and to reduce victim blaming. All primes, data, and syntax for each study can be found on the Open Science Framework (OSF): https://osf.io/t9svu/?view_only=1b20191d88094df596d03e26cce79cfc.

## Pilot

### Method

#### Participants

Participants were recruited from Prolific, an online platform that connects researchers with participants in paid studies. Research suggests that this platform provides high-quality, reliable data, and is considered a better recruitment platform than other online recruitment platforms (e.g., MTurk), as well as specialist participant panels (e.g., Qualtrics; Peer et al., 2022). When signing up to the platform, participants provide a wide range of data that allow researchers to use specific filters to only advertise studies to those who meet the required criteria. Due to the aims of our research, we used the filters to advertise the studies to potential participants who lived in the UK, were 18 years or older, and identified as LGBT+. To maintain the independence of the samples, participants were only able to complete one of the three studies in the research program.

Applying these filters, 131 participants self-identified as LGBT+ and living in the UK completed the study in return for £1.50. Using the primary researcher’s institutional guidance for asking about gender and sexual orientation (Northumbria University, n.d.), which were produced after consultation with leading UK LGBT+ charities (e.g., LGBT Foundation, Stonewall), we asked participants to indicate their gender identification with the question “What term(s) best describe your gender? Please tick all that apply,” and the responses: Man (including trans man)/Woman (including trans woman)/Non-binary/Not listed (please specify)/Prefer not to say. From this, 64 participants identified as men, 60 as women, and 7 people who identified as non-binary. We also asked participants whether they identified as the same or different gender/sex assigned at birth (Yes/No/Prefer not to say). Seven people identified as a different gender/sex than they were assigned at birth, 119 identified as the same gender/sex, and 5 preferred not to say. In addition, sexual orientation was assessed by the question “Which term(s) best describe your sexual orientation?” with the responses: Asexual/Bisexual/Gay/Heterosexual/Lesbian/Queer/Other, please specify/Prefer not to say. More than half identified as bisexual (*n* = 70), 24 as gay, 16 as lesbian, 8 as queer, 7 as pansexual, 4 as asexual, 1 demi-sexual, and 1 biromantic asexual. Ethnicity was assessed using the recommended categories for England and Wales (Office for National Statistics, 2021). The majority of participants identified as White (*n* = 122), 4 as Asian, and 5 as mixed/multiple ethnicities. The ages ranged from 19 to 60 (*M* = 31.34, *SD* = 9.60).

#### Procedure

Initially, the study was an experiment in which participants were randomly assigned to read an adapted BBC article depicting the same homophobic hate crime against two men who were described as either “victims” or “survivors.” The headline read “Men hurt in homophobic attack” and described how two men had been attacked with bottles and endured homophobic verbal abuse outside a pub. The article can be found in the OSF materials. Participants then completed the measures below. However, as the experimental manipulation did not produce any significant differences, but all participants provided their reactions toward the same homophobic hate crime, we collapsed the data across the sample and used the data as a cross-sectional pilot test of the associations outlined above.

#### Materials

Unless specified, scales ranged from 1 (Not at all/ Strongly disagree) to 7 (Very much so/Strongly agree).^2^

*Direct experiences* and *indirect experiences* of hate crimes were assessed as in Paterson et al. (2019a) with participants indicating how many times they had been a *direct* victim of an anti-LGBT+ hate crime within the past 3 years and how many people they knew who had been a victim of an anti-LGBT+ hate crime in the same timeframe. As both measures evidenced positive skew, we treated the variables as dichotomous (0 = no hate experiences vs. 1 = one or more hate experiences).

Participants completed four items referring to the strength of their *LGBT+ identity* including, “I identify with other LGBT+ people,” “I feel good about being LGBT+” (Paterson et al., 2019a; α = .89).

Drawing on Bongiorno et al. (2020), *empathy* was measured with the following stem: “Thinking about the people assaulted in the article that you read, to what extent do you. . .” and four items including “feel empathy for them?” and “feel compassion for them?” (α = .95).

*Victim blame* was measured using the same question stem as above and four items derived from Wakelin and Long (2003) including “they are to blame for the attack?” and “they could have completely avoided what happened” (α = .90).^3^

*Willingness to help* was adapted from Sperry and Siegel (2013) and asked participants to “Suppose you were the head of an organisation that helps people who have been targeted in hate crime. To what extent do you think you would allocate your organisation’s resources to the people in the article in terms of. . .” and specified six different types of help including “emotional support” and “money for legal fees” (α = .83). The measure has been previously used to assess reactions toward rape survivors and has been shown to be a statistically reliable measure (Sperry & Siegel, 2013). Furthermore, because the measure is hypothetical, it is not restricted by participants’ own specific circumstances (e.g., their time and resources) and so taps into a person’s willingness to help—*if* they had the necessary resources, thus providing an insightful measure of helping intentions for this research.

*JWB* was measured using Lipkus et al.’s (1996) 8-item scale including items such as “I feel that the world treats people fairly” and “I feel that people get what they deserve” (α = .87).

### Results

Table 1 presents the descriptive and correlational data for all the measures. It was common for participants to have indirect experiences of hate crime (70%) and these experiences were significantly and positively correlated with direct experiences of hate crime, LGBT+ identity, and empathy. Identification as LGBT+ was strong but not extremely so and was significantly correlated with empathy and willingness to help (positively) and blame (negatively). There was a high level of empathy, a low level of blame, and a moderate level of willingness to help. Empathy was positively and strongly correlated with willingness to help, and these two variables were both negatively correlated with blame. JWB, meanwhile, was significantly and negatively associated with direct experiences of hate crime and empathy, and positively correlated with blame.

**Table 1.** Means, Standard Deviations, and Correlations across the Pilot Study (*N* = 131).

Variable	*M* (*SD*)	2.	3.	4.	5.	6.	7.
1. Indirect experiences	–	.42***	.26**	.21*	−.02	.07	−.07
2. Direct experiences	–	–	.28**	.10	.01	.05	−.19*
3. LGBT+ identity	4.83 (1.37)	–	–	.31***	−.28***	.21*	−.15
4. Empathy	6.23 (1.00)	–	–	–	−.53***	.54***	−.18*
5. Blame	2.13 (1.20)	–	–	–	–	−.38***	.31***
6. Willingness to help	4.86 (1.04)	–	–	–	–	–	−.20*
7. JWB	2.95 (1.14)	–	–	–	–	–	–

*Note.* 70% of participants had indirect experiences of hate crime and 35% had direct experiences of hate crime in the past 3 years. JWB = just world beliefs.

* *p* < .05. **p < .01. **p < .001.

#### Mediation analyses

**Figure 1.**
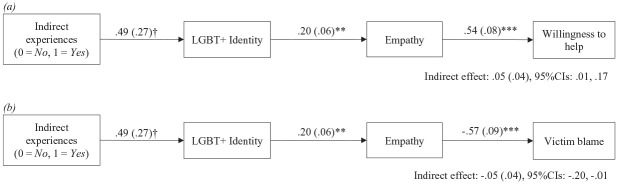
Serial mediation tests of indirect experiences of hate crime via LGBT+ identity and empathy on (a) willingness to help victims and (b) victim blame, controlling for direct experiences of hate crime and JWB (Pilot Study *N* = 131). *Note.* 1,000 bootstrap samples using PROCESS Model 6. JWB = just world beliefs. †*p* = .07. **p < .01. ***p < .001.

Using PROCESS model 6, with 95% confidence intervals (CIs) and 1,000 bootstrap samples (Hayes, 2017), we tested two serial mediation models in which indirect experiences of hate crimes were set as the predictor, LGBT+ identity as the first mediator leading to empathy as the second mediator, and then either (a) willingness to help or (b) blame as the outcome (Figure 1). Direct experiences of hate crimes and JWB were controlled for in both models. As shown in Figure 1, both serial mediations were significant. Indirect experiences of hate crimes were positively associated with LGBT+ identification which serially predicted greater empathy which was then positively related to willingness to help (indirect effect = 0.05, *SE* = 0.04, 95% CIs [0.01, 0.17]) and negatively related to victim blame (indirect effect = −0.05, *SE* = 0.04, 95% CIs [−0.20, −0.01]).

Together, the serial mediations provide initial evidence that indirect experiences of hate crime strengthen individuals’ LGBT+ identity which leads to more empathy for hate crime victims and, thus, less blame and more willingness to help. Nevertheless, as the findings came from collapsing data across experimental conditions and from a relatively small sample size, we sought to replicate the findings with a cross-sectional study involving a much larger sample size in Study 1.

## Study 1

### Method

#### Participants

The required minimum sample size was calculated by the linear multiple regression function in G*Power (Faul et al., 2007) specifying three tested predictors (*indirect experiences, LGBT+ identity*, and *empathy*), five total predictors (*direct experiences* and *JWB* as covariates), and using 80% power, α = .05, and a small effect size (*f*^2^ = 0.02 which was derived from the lowest correlation coefficient among the variables of interest in the pilot study *r* = 0.21). This suggested a minimum sample size of 550 and, accounting for possible attrition and invalid data, we recruited 600 participants from Prolific in return for £1.25.

Using the same demographic measures as the Pilot, all participants identified as LGBT+, 372 identified as women, 173 as men, 47 as non-binary, 5 as agender, and 3 preferred not to say. In all, 55 identified as a different gender/sex than they were assigned at birth, and 545 identified as the same gender/sex. More than half identified as bisexual (*n* = 313), 85 as gay, 65 as lesbian, 46 as queer, 25 as asexual, 22 as pansexual, 17 as heterosexual, 6 as questioning, and 21 preferred not to say. The majority identified as White (*n* = 525), 29 as having multiple/mixed ethnicities, 24 as Asian, 15 as Black, 6 as Arab, and one preferred not to say. The average age was 30.86 (*SD* = 11.05, range = 18–74).

#### Procedure and materials

All participants read a short and ostensibly real newspaper article depicting a homophobic assault with the headline “Man’s leg broken in a violent homophobic hate attack.” An example of the article can be found in the OSF materials. A manipulation check showed that all participants correctly identified that the assault was described as a homophobic hate crime. Participants then completed the same measures as in the pilot study: *indirect experiences* and *direct experiences* of hate crime (these again showed skewed responses so were dichotomized where 0 = no experiences and 1 = one or more experiences), *empathy* (α = .93), *blame* (α = .73), *willingness to help* (α = .80), and *JWB* (α = .88).

To further explore how the different facets of LGBT+ identity may be associated with reactions to hate crimes, in this study we measured *LGBT+ identity* with the 27-item Lesbian, Gay, and Bisexual identity scale (Mohr & Kendra, 2011). As we hypothesized the relatively more positive aspects of the LGBT+ identity would most likely be associated with both indirect experiences of hate and empathy, we focused on the two subscales of *Identity affirmation* (3 items, e.g., “I am glad to be an LGB+ person,” α = .91) and *Identity centrality* (5 items e.g., “Being an LGB+ person is a very important aspect of my life”).^4^

### Results

Table 2 shows that the descriptive and correlations for the variables in Study 1 are strikingly similar to those in the pilot study. Indirect experiences of hate crimes were frequent (68%) and nearly a third of participants (30%) had been directly victimized in a hate crime in the past 3 years. Indirect experiences and direct experiences of hate crimes were again significantly and positively correlated with one another, as well as being significantly associated with a stronger LGBT+ identity (both in terms of identity affirmation and identity centrality), and greater empathy for the victim. On average, participants were happy with their LGBT+ identity (*identity affirmation*) and the identity was moderately central to their life (*identity centrality*). Both these aspects of LGBT+ identity were significantly associated with greater empathy for the victim and greater willingness to help the victim, as well as less victim blame. Replicating the pilot study, there was considerable empathy for the victim, very little victim blame, and a general willingness to help the victim. Empathy and willingness to help were again strongly positively correlated with one another and were negatively correlated with victim blame. JWB was significantly and negatively correlated with indirect and direct experiences of hate crimes, identity affirmation, empathy, and willingness to help, and positively correlated with victim blame.

**Table 2.** Means, Standard Deviations, and Correlations across Study 1 (*N* = 600).

Variable	*M* **(*SD*)**	2.	3.	4.	5.	6.	7.	8.
1. Indirect experiences	–	.34***	.29***	.21***	.13***	.01	.08	−.12**
2. Direct experiences	–	–	.20***	.23***	.08*	−.07	.05	−.12**
3. Identity affirmation	5.25 (1.53)	–	–	.52***	.27***	−.14***	.21***	−.11**
4. Identity centrality	3.63 (1.52)	–	–	–	.19***	−.12**	.16***	−.06
5. Empathy	6.68 (0.67)	–	–	–	–	−.37***	.33***	−.16**
6. Blame	1.14 (0.39)	–	–	–	–	–	−.14***	.17***
7. Willingness to help	5.25 (0.97)	–	–	–	–	–	–	−.15***
8. JWB	2.99 (0.94)	–	–	–	–	–	–	–

*Note.* 68% of participants had indirect experiences of hate crime and 30% had direct experiences of hate crime in the past 3 years. JWB = just world beliefs.

* *p* < .05. **p < .01. **p < .001.

#### Mediation analyses

Replicating the Pilot study, serial mediation models were analyzed using PROCESS model 6 with 95% CIs and 1,000 bootstrap samples (Hayes, 2017). Indirect experiences of hate crime were the predictor, identity affirmation or identity centrality was the first mediator (with the other being used as a covariate), empathy was the second mediator, and blame or willingness to help was the outcome. Direct experiences of hate crime and JWB were used as covariates in the four models.

**Figure 2.**
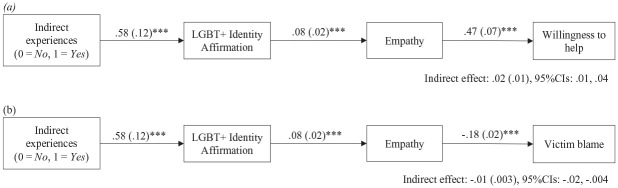
Serial mediation tests of indirect experiences of hate crime via LGBT+ identity affirmation and empathy on (a) willingness to help victims and (b) victim blame, controlling for direct experiences of hate crime, JWB, and centrality of LGBT+ identity (Study 1 *N* = 600). *Note.* 1,000 bootstrap samples using PROCESS Model 6. JWB = just world beliefs. †*p* = .07. **p < .01. ***p < .001.

Figure 2 shows the two significant serial mediations which included identity affirmation as the first mediator: indirect experiences were positively associated with identity affirmation which, in turn, was positively associated with empathy which (in separate models) was positively associated with (a) willingness to help (indirect effect = 02, *SE* = 0.01, 95% CIs [0.01, 0.04]) and (b) was negatively associated with blame (indirect effect = −0.01, *SE* = 0.003, 95% CIs [−0.02, −0.004]). The same mediation models were then conducted with identity centrality as the first mediator and identity affirmation as a covariate. When controlling for identity affirmation, however, indirect experiences of hate crime were no longer significantly associated with identity centrality (*b* = 0.07, 95% CIs [−0.17, 0.31]) and so the indirect effects were not significant (willingness to help *b* = 0.001, 95% CIs [−0.001, 0.008], blame *b* = −0.0003, 95% CIs [−0.002, 0.0005]).

## Study 2

Corroborating the findings of the Pilot study, Study 1 suggests that indirect experiences of hate crimes enhance positive feelings toward being LGBT+ (but not necessarily the centrality of the LGBT+ identity) which, in turn, leads to greater ingroup empathy, resulting in less victim blaming and more willingness to help to ingroup victims. Nevertheless, the data remain correlational and so claims of causality are problematic. In Study 2, therefore, we aimed to experimentally assess how indirect experiences of hate-motivated crimes (vs. non-hate-motivated crimes) influence individuals’ reactions to victims. In addition, acknowledging that the strength of LGBT+ identification is a relatively enduring trait (Jaspal, 2022) and so not easily manipulated in short experiments, we used the group membership of the victim (LGBT+ vs. heterosexual) to assess how participants’ LGBT+ identity further impacted on their reactions to victims.

In doing so, we hypothesized that participants reading about an anti-LGBT+ hate motivated crime would report greater empathy, greater willingness to help, and less victim blame than participants who read about the same crime which was described as a case of mistaken identity (non-hate crime). Furthermore, when the victim was portrayed as gay (LGBT+ victim) there would be greater empathy, greater willingness to help, and less blame than when the victim was portrayed as heterosexual. In addition, an interaction effect was expected in which crimes that contained any element of an ingroup attack (i.e., was hate motivated and/or against a gay person) would elicit greater empathy, greater willingness to help, and less victim blame than the crime that was not against the ingroup (i.e., the non-hate motivated attack on a heterosexual person). Finally, replicating the cross-sectional data, we hypothesized that the experimental effects on victim blame and willingness to help would be mediated by empathy.

### Method

#### Participants

Using G*Power (Faul et al., 2007) specifying a 2 × 2 analysis of covariance (ANCOVA) with four groups, three covariates, 80% power, α = .05, and a small effect size (*f*^2^ = 0.01), the minimum recommended sample size was 787. Expecting some attrition but also financially limited, we initially recruited 801 participants from Prolific to participate in the online study in return for £1.25. In total, 65 participants were excluded for failing the manipulation check(s) and 79 for not identifying as LGBT+, leaving 657 participants. Using the same demographic questions as before, 429 identified as women, 160 as men, 65 as non-binary, and three preferred not to say. In all, 58 participants indicated that their gender identity was not the same as their gender/sex assigned at birth and 599 indicated that their gender/sex was the same. More than half identified as bisexual (*n* = 370), 86 lesbian, 74 gay, 42 queer, 31 asexual, 24 pansexual, 2 questioning, 1 heterosexual, and 27 did not specify. Most participants identified as White (*n* = 568), 41 mixed/multiple ethnicities, 36 Asian, 10 Black, 1 Arab, and 1 not specified. The average age was 28.25 (*SD* = 10.29, range = 18–70).

#### Procedure

Participants were randomly allocated to read one of four near-identical articles depicting a physical assault against a man in which the crime was described as a hate crime or a case of mistaken identity (*motive*: hate vs. non-hate), and the article referred to the man’s husband or wife which served to infer the victim’s LGBT+ identity (*group*: ingroup vs. outgroup). An example of the article is presented in the OSF materials.

#### Materials

*Indirect* and *direct experiences* of hate crime (both dichotomized), e*mpathy* (α = .91), *blame* (α = .60), *willingness to help* (α = .80), and *JWB* (α = .87) were measured as in the two previous studies, and we used the four-item *LGBT+ identity* (α = .88) as we had in the Pilot.

### Results

Across the sample, 71% of participants had indirect experience of hate crimes, 37% had direct experience of hate crimes, participants had a moderate level of LGBT+ identity (*M* = 4.70, *SD* = 1.38), and a low-level belief in a just world (*M* = 2.65, *SD* = 0.90). A series of χ^2^ tests and a 2(*motive*: hate vs. non-hate) × 2(*group*: ingroup vs. outgroup) multivariate analysis of variance (MANOVA) revealed that these covariates did not differ across experimental conditions: LGBT+ identity *F*(3,653) = 0.70, *p* = .55; JWB *F*(3,653) = 0.31, *p* = .82; indirect and direct experiences of hate crimes across conditions (all χ^2^s(1) < 0.68, all *p*s < 0.42).

Table 3 reports the 2(*motive*: hate vs. non-hate) × 2(*group*: ingroup vs. outgroup) MANCOVA on the dependent variables of empathy, blame, and willingness to help, controlling for indirect and direct experiences of hate crime, JWB, and LGBT+ identity. As hypothesized, there were significant main effects of *motive* on empathy and willingness to help, with Bonferroni comparisons indicating that hate-motivated crimes elicited more empathy (*M*_hate_ = 6.70 vs. *M*_non-hate_ = 6.43, *p* < .001) and a greater willingness to help (*M*_hate_ = 5.24 vs. *M*_non-hate_ = 5.09, *p* = .05). However, there was no main effect of *motive* on blame (*p* = .24).

**Table 3.** Study 2: Means and Standard Deviations of Experimental Groups and Two-Way MANCOVA Controlling for Hate Crime Experiences, JWB, and LGBT+ Identity.

Variable	Ingroup hate (*n* = 159) *M* (*SD*)	Outgroup hate (*n* = 172) *M* (*SD*)	Ingroup non-hate (*n* = 157) *M* (*SD*)	Outgroup non-hate (*n* = 169) *M* (*SD*)	Main effect of motive *F*(1,649), $\eta_{p}^{2}$	Main effect of group *F*(1,649), $\eta_{p}^{2}$	Interaction of motive x group *F*(1,649), $\eta_{p}^{2}$
Empathy	6.75 (0.53)	6.66 (0.60)	6.56 (0.63)	6.29 (0.83)	28.97***, $\eta_{p}^{2}=0.04$	11.85***, $\eta_{p}^{2}=0.02$	2.74†, $\eta_{p}^{2}=0.004$
Blame	1.14 (0.30)	1.18 (0.34)	1.16 (0.33)	1.22 (0.35)	1.40, $\eta_{p}^{2}=0.00$	3.84*, $\eta_{p}^{2}=0.01$	0.18, $\eta_{p}^{2}=0.00$
Willingness to help	5.34 (1.01)	5.14 (0.91)	5.16 (0.89)	5.00 (1.06)	3.86*, $\eta_{p}^{2}=0.00$	5.84*, $\eta_{p}^{2}=0.01$	0.57, $\eta_{p}^{2}=0.00$

*Note.* JWB = just world beliefs.

† *p* < .10. *p < .05. **p < .01. **p < .001.

Table 3 also reveals that there were significant main effects of *group* membership of the victim on all three dependent variables. In line with expectations, Bonferroni comparisons showed that ingroup members elicited greater empathy (*M*_ingroup_ = 6.65 vs. *M*_outgroup_ = 6.48, *p* = .001), greater willingness to help (*M*_ingroup_ = 5.25 vs. *M*_outgroup_ = 5.07, *p* = .02), and less blame (*M*_ingroup_ = 1.15 vs. *M*_outgroup_ = 1.20, *p* = .05).

In addition to the significant main effects, the interaction effect of *motive* and *group* on empathy was approaching significance (*p* = .10). As hypothesized, post hoc comparisons revealed that outgroup victims in non-hate crimes received significantly less empathy than the other three experimental conditions which all included at least one intergroup aspect (all *p*s < 0.001). Ingroup victims in hate crimes received significantly more empathy than ingroup victims in non-hate crimes (*p* = .01), but the difference between ingroup victims and outgroup victims in hate crimes was not significant (*p* = .21).

#### Mediation analyses

**Figure 3.**
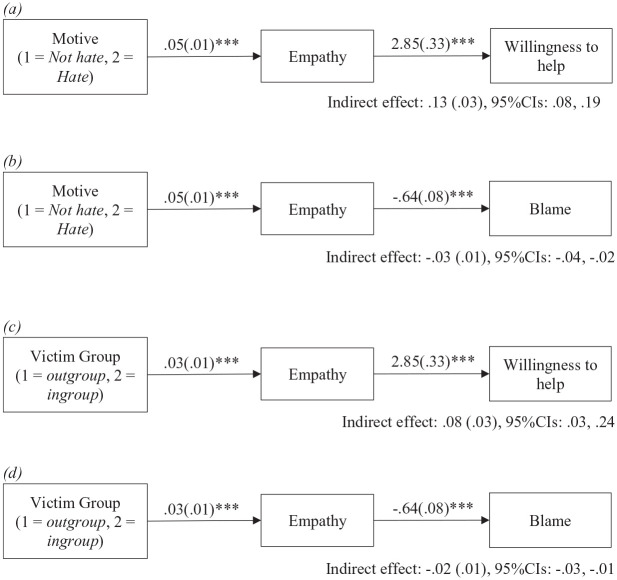
Mediation tests of how the experimental manipulations influenced willingness to help victims and victim blame via empathy, controlling for direct and indirect experiences of hate crime, and JWB (Study 2 *N* = 657). *Note.* 1,000 bootstrap samples using PROCESS Model 4. JWB = just world beliefs. ***p < .001.

To examine whether the experimental effects on blame and willingness to help were mediated by increased empathy, four mediation models were conducted using PROCESS model 4, 95% CIs, and 1,000 bootstrap samples (Hayes, 2017). The predictor variable was either *motive* or *group* (with the other experimental manipulation being used as a covariate), the mediator was empathy, the outcome was either blame or willingness to help, and the covariates were JWB, LGBT+ identity, and both direct and indirect experiences of hate crime. Figure 3 shows that the proposed indirect effects were all significant. Compared to non-hate motivated crimes, hate crimes elicited more empathy which, in turn, was associated with (a) a greater willingness to help (indirect effect: 0.13, *SE* = 0.03, 95% CIs [0.08, 0.19]) and (b) less victim blame (indirect effect: −0.03, *SE* = 0.01, 95% CIs [−0.04, −0.02]). Similarly, ingroup victims received more empathy than outgroup victims and this empathy was again associated with (c) greater willingness to help (indirect effect: 0.08, *SE* = 0.03, 95% CIs [0.03, 0.24]) and (d) less victim blame (indirect effect: −0.02, *SE* = 0.01, 95% CIs [−0.03, −0.01]).

These findings suggest that hate-motivated crimes elicit less victim blame and a greater willingness to help because LGBT+ people feel more empathic toward people (irrespective of whether they are ingroup or outgroup members) who have been targeted in a crime motivated by prejudice toward their identity group. Similarly, crimes involving ingroup members (irrespective of their motivation) elicit less victim blame and a greater willingness to help than crimes involving outgroup members because LGBT+ participants exhibit an ingroup empathy bias. Of note, all these mediations are significant while controlling for the other experimental manipulation, thereby showing the unique effects of both *motive* (a symbolic attack against the group identity) and *group* (an attack on a fellow LGBT+ person) on participants’ reactions.

## General Discussion

Across a coherent program of research involving three studies, we show that ingroup responses to anti-LGBT+ hate crime victims are overwhelmingly positive and protective. We consistently find that fellow LGBT+ people report high levels of empathy, strong helping intentions, and low levels of victim blame. In the Pilot Study and Study 1, we show that these reactions stem from indirect experiences of hate crimes, serially mediated by LGBT+ identity and empathy. That is, knowing victims of hate crimes was associated with a stronger LGBT+ identity which, in turn, was associated with greater empathy for the victim that was then associated with greater willingness to help and less victim blame. Study 2 provided further experimental evidence for this mechanism and highlighted the importance of the group aspects of hate crimes. Crimes that involved a hate motivation and/or an ingroup member elicited greater empathy which, in turn, promoted a greater willingness to help the victim and attribute less blame to the victim. Together, the findings provide cogent evidence that the LGBT+ community is generally a principal source of support for victims of anti-LGBT+ hate crimes, and this support can be best understood by integrating literature from different disciplines.

The research contributes to existing theoretical knowledge in numerous meaningful ways. The findings provide further support for the utility of understanding the impacts of hate crime through the lens of SCT (Turner et al., 1987) and IET (Smith, 1993). Expanding upon previous research, we show that these group-based crimes not only motivate ingroup empathic responses (Paterson et al., 2019a), but they also impact both helping and blaming reactions and do so because the crimes target two unique but related facets of the social group: its group members *and* its group identity. By targeting both these aspects, hate crimes are clear examples of group-based hostility that significantly impacts a range of emotions and reactions that can be explained by group-based theories.

Acknowledging the meaningful role of group identity, we make a further unique contribution to the literature by clarifying the specific aspects of the LGBT+ identity that are associated with responses to victims. Notably, Study 1 reveals that indirect experiences of hate crime are positively linked to both the centrality of the LGBT+ identity (e.g., the importance of the LGBT+ identity to the individual) and the affirmation of the LGBT+ identity (e.g., being glad to be an LGBT+ person). Both these aspects of identity were correlated with greater empathy and less victim blame; however, mediation analyses revealed that LGBT+ identity affirmation was the strongest predictor of empathy, while the associations of identity centrality became nonsignificant. This suggests that indirect experiences of hate crimes influence many aspects of the LGBT+ identity, but it is the pride of being LGBT+ that is instrumental in shaping reactions to victims, including empathic and helping reactions.

Across the three studies, we provide remarkably consistent evidence that empathy is associated with both a greater willingness to help the victim and less victim blame. This provides support for the empathy-altruism hypothesis (Batson, 1991) and is particularly notable because it is the first to show the psychological mechanisms underpinning ingroup helping and blaming reactions to victims in hate crime contexts.

Moreover, by highlighting the links between empathy, willingness to help, and victim blame, we advance a more nuanced perspective on *intergroup empathy bias*. Previous research has invariably focused on the negative implications of this bias, for example, highlighting how ingroup empathy bias typically translates to *less* empathy for outgroup members which can perpetuate and even exacerbate intergroup conflict (Cikara et al., 2011). However, the present research shows that ingroup empathy bias should not always be thought of as negative in outcome. Indeed, in Study 2, the average empathy for an outgroup victim in a non-hate crime was the lowest of all conditions but was still extremely high (*M* = 6.29 out of 7). This suggests that LGBT+ people are highly empathic toward *all* victims, regardless of their group identity, thus indicating that the ingroup empathy bias does not necessarily translate to the detriment of outgroup members. Instead, considering the LGBT+ community continues to be stigmatized and discriminated against (Home Office, 2021), the relative enhanced empathy shown toward hate crime victims may be more appropriately characterized as a necessary, group-based defense mechanism, motivating more help and support when other avenues are thwarted. We believe this more nuanced understanding of ingroup biases and their impacts—especially for minority groups—adds to the understanding of intergroup empathy bias.

The current findings also contribute to the victim blame literature. Previous research has generally focused on outgroup members’ blaming tendencies (e.g., Rayburn et al., 2003), or has provided qualitative evidence for ingroup victim blaming in hate crimes (e.g., Bell & Perry, 2015). To the best of our knowledge, we are the first to show that LGBT+ individuals blame ingroup members *less* than outgroup members when targeted in the same type of crime (Study 2). From a theoretical standpoint, this is important for two reasons. First, we provide further clarity on ingroup victim blaming in hate crime by showing that although ingroup victim blaming does occur, thus supporting Bell and Perry’s (2015) findings, it does so at minimal levels and, importantly, ingroup members receive comparatively *less* blame than outgroup members. Second, as the analyses statistically controlled for the powerful influence of JWB, the findings highlight the importance of group identities in victim blaming—and do so above and beyond powerful worldviews.

### Implications

We have shown that individuals identifying as LGBT+ share important emotional and empathic bonds that engender positive and protective reactions toward other group members in the aftermath of targeted victimization. Considering the marginalizing consequences of hate crimes (Walters, 2022), it is perhaps unsurprising that LGBT+ people coalesce to provide the support that they may not always receive from beyond the confines of the community’s protective walls (see Godzisz & Mazurczak, 2022). Previous research has explained how LGBT+ people (and other commonly targeted groups) often feel connected through a sense of “shared suffering” that is constantly reinforced by the symbolic messages invoked by hate crimes (Walters, Paterson, McDonnell, et al., 2020). This study has shown that LGBT+ members are likely to respond to the *in terrorem* effect of hate crimes in ways that attempt to buffer their harmful effects while promoting a greater sense of belonging in the LGBT+ community.

The empathic reactions from LGBT+ community members have implications for policy and practice. By evidencing the salience of group identities, the research underscores the importance of resourcing LGBT+ organizations and charities to support hate crime victims. These organizations remain important conduits through which LGBT+ victims can access safe spaces and where LGBT+-sensitized support can be offered. However, funding such organizations must not detract from the responsibility of public policymakers to improve criminal justice responses to anti-LGBT+ hate crimes. Presently, there are a growing number of anti-LGBT+ hate crimes in the UK and elsewhere (e.g., Home Office, 2021), and LGBT+ people regularly report being mistrustful of, and less satisfied with, the criminal justice system (CJS; Walters, Paterson, Brown, et al., 2020). It is clear, then, that policymakers need to continue to improve internal systems to tackle hate crimes while offering targeted services that specifically aim to support marginalized groups when they interact with different agencies within the CJS (e.g., Haynes et al., 2023). Drawing on the current findings, policymakers ought to give priority to strategies and measures that utilize shared group identities. For example, procedural rights could be enhanced via the (re)introduction of enhanced training around hate crimes for all officers, as well as specially trained liaison officers. This will likely help to promote empathy between victims and criminal justice personnel, which, in turn, will increase confidence and satisfaction with the CJS. Service rights can also be enhanced where local provision is spread across service providers, such as where victim support is offered both by organizations such as Victim Support (the UK’s general victims support service) and LGBT+ charities such as GALOP (the UK’s LGBT+ anti-abuse charity).

### Limitations

A limitation of the current research is the operationalization of the LGBT+ community as one homogeneous group. This superordinate definition of the community overlooks the diversity of the community, the different experiences of subgroups (Walters, Paterson, Brown, et al., 2020), and the tensions that may arise between them (e.g., Welzer-Lang, 2008). Furthermore, as our samples were overwhelmingly White and cisgender, we did not have the statistical power to examine important intersectional issues, including how ethnicity and gender identity may have further impacts on participants’ responses. However, as previous research has shown that LGBT+ people from minority ethnic backgrounds fear hate crimes to a greater extent than majority ethnic group members (Rogers et al., 2023), and trans people experience more—and are impacted more—by hate crimes (Walters, Paterson, Brown, et al., 2020), the current research is likely to underestimate the reactions of people who are marginalized across multiple identities. In addition, replicating previous research (Paterson et al., 2019a), we elicited people’s reactions to a gay man in a hate crime. These factors all limit the generalizability of the findings. Future research would be well placed to build upon the current findings to further explore the impacts of intersectionality and examine whether victims from other subgroups (e.g., lesbian woman, bisexual man) and with intersecting identities (e.g., various ethnicities, gender identities, sexual orientations) elicit different reactions from the diverse LGBT+ subgroups. With that said, our previous research has shown that the impacts of anti-LGBT+ hate crimes are experienced to a similar extent across some of the subgroups that make up LGBT+ communities (Paterson et al., 2018).

We hope that this study creates opportunities for other researchers to develop our measures. Replicating previous research (Bell & Perry, 2015), we found that victim blame was extremely low, especially in Studies 1 and 2, which could explain the relatively small effect sizes for the measure, along with the lack of significant difference of motive in victim blame in Study 2. Experimental primes that incorporate greater ambiguity on the role and responsibility of the victim (e.g., having the victim start the altercation) may provide more variance in responses, thereby avoiding statistical floor effects and would help to better understand potential differences in victim blame. In addition, there are opportunities to further develop the willingness to help measure. Although the measure is useful for understanding participants’ hypothetical intentions to help victims and provide insights into how the community would like victims of these crimes to be compensated, such intentions may not translate to actual behaviors in real life. Indeed, the measure asks participants to allocate an agency’s resources which would not incur any personal cost. As the costs and benefits of helping victims is a potent predictor of helping (Hao et al., 2023), future longitudinal research could adopt methods that capture the cost/benefit analysis and actual individual-level behaviors, taking care to control for extraneous variables (e.g., variation in participants’ time and resources) and ethical considerations (e.g., taking time and money from participants).

## Conclusion

As hate crimes continue to blight individuals, communities, and societies as a whole, this research provides important advances to our theoretical knowledge of how affected communities react to such incidents. We show that in the face of stigmatization and violence, LGBT+ communities are highly likely to provide empathic and supportive responses to victims that are rooted in a shared superordinate sexual and gender identity. These findings advance a range of interdisciplinary literature and theories (e.g., SCT, IET, victim blame), and highlight the practical importance of LGBT+-focused responses to supporting victims of hate crime.

## Notes

1. Although not a focus of this paper, the analyses of the association between JWB and reactions toward hate crime in the three studies are provided in the supplementary materials on the OSF https://osf.io/t9svu/?view_only=1b20191d88094df596d03e26cce79cfc.

2. As part of the planned experiment, participants also completed an emotional Stroop test (Hafer, 2000) but this is not reported here as it does not relate to the hypotheses.

3. Empathy and blame evidenced high levels of negative and positive skew, respectively. We naturally log-transformed the data and conducted the analyses but as the transformed and untransformed data produced the same results, we present the untransformed data here for ease of interpretation.

4. The data and analysis for the other subscales can be found in the OSF files: https://osf.io/t9svu/?view_only=1b20191d88094df596d03e26cce79cfc
